# Acute epididymo-orchitis complicated by outcomes of either testicular necrosis or complete recovery: Two case reports

**DOI:** 10.1097/MD.0000000000042391

**Published:** 2025-05-02

**Authors:** Bin Cao, Chaohong Zhang, Fan Xiao, Yuye Wu, Qiufang Ouyang

**Affiliations:** a Ultrasound Department, The Second Affiliated People’s Hospital of Fujian University of Traditional Chinese Medicine, Fuzhou, Fujian, China; b Urology Department, The Second Affiliated People’s Hospital of Fujian University of Traditional Chinese Medicine, Fuzhou, Fujian, China.

**Keywords:** case report, complication, epididymo-orchitis, orchiectomy, testicular necrosis

## Abstract

**Rationale:**

Acute epididymo-orchitis, a common urological emergency requiring prompt intervention to prevent complications like testicular ischemia. This study highlights the use of serial Doppler ultrasound monitoring in patients with acute epididymo-orchitis, particularly in high-risk individuals.

**Patient concerns:**

Case 1: A 70-year-old male with a history of bladder cancer, prostate malignancy, and hypertension, presented with scrotal swelling, pain, and lower abdominal discomfort. Physical examination revealed an enlarged and tender right testicular epididymis, with normal findings on the left. Ultrasound showed increased blood flow to the right testicular epididymis, indicating inflammation. Case 2: A 34-year-old male presented with scrotal swelling, pain, and lower abdominal discomfort. Ultrasound revealed increased testicular and epididymal blood flow, suggesting inflammation. The antibiotic therapy was adjusted according to the continuous ultrasound monitoring.

**Diagnoses:**

Both cases were ultimately diagnosed as testicular epididymal inflammation.

**Interventions:**

Patient 1 underwent anti-inflammatory therapy and orchiectomy. Patient 2 was treated with antibiotic therapy and recovered.

**Outcomes:**

Patient 1 experienced testicular necrosis, whereas Patient 2 achieved a full recovery.

**Lessons:**

The importance of serial Doppler ultrasonography: delayed follow-up imaging in this case allowed ischemic changes to progress irreversibly, despite initial Doppler findings showing increased perfusion. Early and repeated imaging is critical to monitor disease progression and guide timely interventions. Limitations of inflammatory markers: the disease worsened although the patient’s leukocytosis and IL-6 levels improved markedly during treatment, highlighting that relying solely on blood tests is insufficient to determine treatment efficacy. Patient-specific risk stratification: high-risk individuals require more aggressive diagnostic and therapeutic protocols to prevent irreversible complications.

## 1. Introduction

Acute epididymo-orchitis (EO) is a common urological emergency characterized by inflammation of the epididymis and testis, typically presenting with scrotal pain, swelling, and systemic symptoms. While antibiotics therapy is the mainstay of treatment, a subset of patients may progress to severe complications such as testicular necrosis, necessitating orchiectomy.^[1]^ While scrotal Doppler ultrasound are routinely employed to evaluate perfusion and rule out torsion, their potential for serial quantitative monitoring remains underexplored in clinical workflows.^[2]^ Additionally, reliance on inflammatory biomarkers (e.g., leukocytosis, interleukin-6 [IL-6]) as surrogates for disease severity or treatment response carries inherent risks, as these markers may not always correlate with the pathology at the local tissue level.^[3]^

This study addresses 2 pivotal, yet under recognized, challenges in acute EO management. First, we propose a novel application of serial Doppler ultrasound by leveraging the contralateral healthy testicle as an internal control to quantify perfusion asymmetry and track blood flow changes pre and posttreatment. This method offers an objective, reproducible framework to guide clinical decisions – a paradigm not yet integrated into existing guidelines. Second, we critically examine the limitations of biomarker-driven clinical judgment, illustrated by a case where inflammatory markers have improved despite worsened EO, underscoring the potential for misinterpretation. By bridging these evidence gaps, our findings advocate for a more frequent, Doppler ultrasound-supported approach to optimize outcomes in EO management.

## 2. Case presentation

This article analyzes 2 patients with acute epididymo-orphitis (EO) who exhibited markedly contrasting clinical trajectories: one progressing to testicular necrosis despite intervention, and the other achieving full recovery through adaptive treatment adjustments based on ultrasound data. Below is a detailed analysis of Case 1 and 2.

### 2.1. Case 1: testicular necrosis and orchiectomy

A 70-year-old male with a complex medical history – including bladder cancer, prostate malignancy, and hypertension – presented to the urology department on June 1, 2024, with a 3-day history of progressive right scrotal swelling, pain, and ipsilateral lower abdominal discomfort. Physical examination revealed marked tenderness and swelling of the right epididymis, with no abnormalities on the contralateral side.

**Figure 1.**
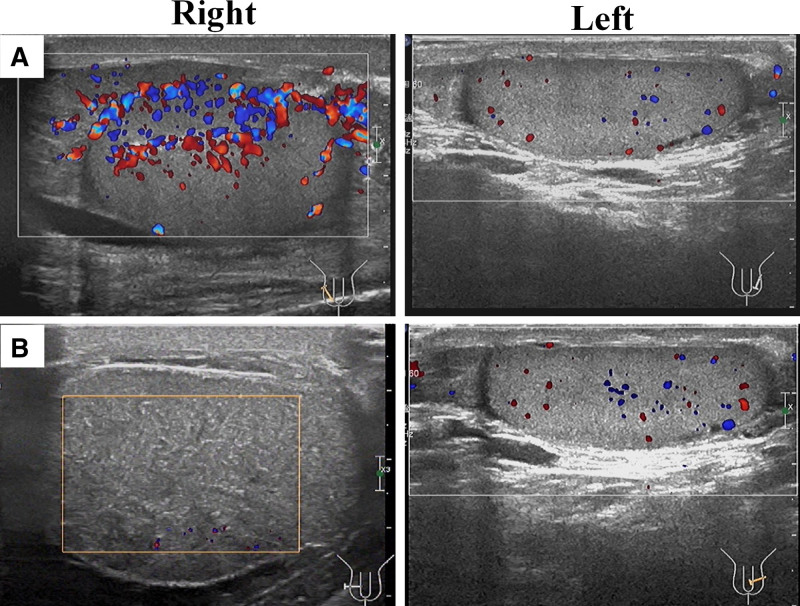
Scrotal ultrasound images in a 70-yr-old patient with scrotal pain. (A) Initial color Doppler ultrasonography reveals markedly increased blood flow in the right testis as compared to the physiological perfusion patterns in the left testis. (B) Day-11 follow-up ultrasound reveals disappeared perfusion in the right testis relative to the normal vascularity in the left testis.

Initial imaging (June 3, 2024): Doppler ultrasonography demonstrated significantly increased blood flow in the right testis compared to the left (Fig. 1A), consistent with acute EO. Hematologic (June 3, 2024): Leukocytosis (white blood cells [WBC] 34.41 × 10⁹/L), neutrophils 85%, and markedly elevated IL-6 (6129.94 pg/mL), indicative of systemic inflammation. Urinalysis (June 3, 2024): Hematuria, pyuria (78.54 WBC/µL), leukocyte esterase, and altered specific gravity. Subsequent urine culture (June 5, 2024) confirmed *Escherichia coli* infection.

**Figure 2.**
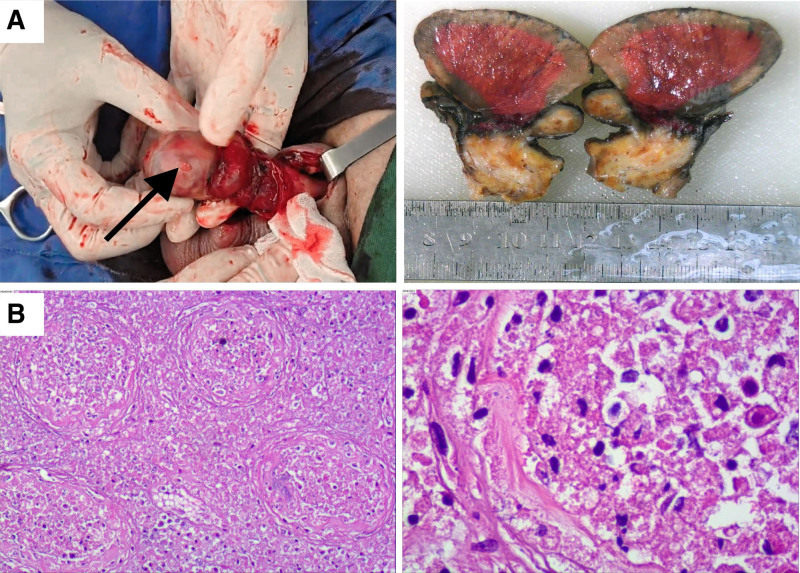
Gross and histopathologic features of testicular infarction in the patient with acute epididymo-orchitis. (A) Intraoperative gross specimen demonstrating ischemic pallor. There is no bleeding upon compression (arrow). Cross-sectional view reveals a gray-white peripheral zone with central gray-red discoloration. (B) Low-power (10×) hematoxylin-eosin staining showed extensive coagulative necrosis of testicular parenchyma (left). High-power magnification (200×) highlighted neutrophilic infiltration, with pyknotic nuclei and eosinophilic cytoplasmic changes (right).

The patient was started on Piperacillin/Tazobactam (4.5 g, intravenously administered every 8 hours). Follow-up blood tests on June 9, 2024, showed improved leukocytosis (WBC: 13.38 × 10⁹/L) and IL-6 (678.52 pg/mL). Hematuria, pyuria (76.23 WBC/µL), leukocyte esterase. A follow-up scrotal ultrasound (June 11, 2024) identified worsening right testicular ischemia (Fig. 1B), characterized by diminished parenchymal perfusion and epididymal hyperemia. Due to concerns for irreversible compromise, emergency right testicular exploration was performed after family consultation. Intraoperatively, we found the right testis was dusky red, firm, and nonpitting, with thickened tunica vaginalis. The epididymis was enlarged, irregular, and nontender. No torsion was noted. Intraoperative interventions (lidocaine infiltration of the spermatic cord, warm saline irrigation) failed to restore perfusion. A parenchymal incision yielded no blood, confirming necrosis (Fig. 2A). Postoperative histopathology confirmed testicular infarction and epididymitis, with histologic findings of focal lymphocytic, neutrophilic, and histiocytic infiltration, along with purulent abscesses (Fig. 2B). The patient recovered uneventfully, receiving postoperative cefepime (1 g every 12 hours) and oral antibiotics.

### 2.2. Case report 2：complete recovery through ultrasound-guided therapeutic adaptation

A 34-year-old male presented on August 13, 2023, with a 3-day history of progressive right scrotal pain, swelling, and intermittent fever (peak 38.9°C). On admission, the temperature is 37.9°C. The right testicle was enlarged, firm, and nodular, exhibiting pronounced tenderness. The overlying scrotal skin was erythematous, with the demarcation between the right testicle and epididymis being indistinct. There was notable edema involving the right scrotal and inguinal regions, and palpation of the tender right spermatic cord was impeded. The left testicle and epididymis appeared normal on examination.

**Figure 3.**
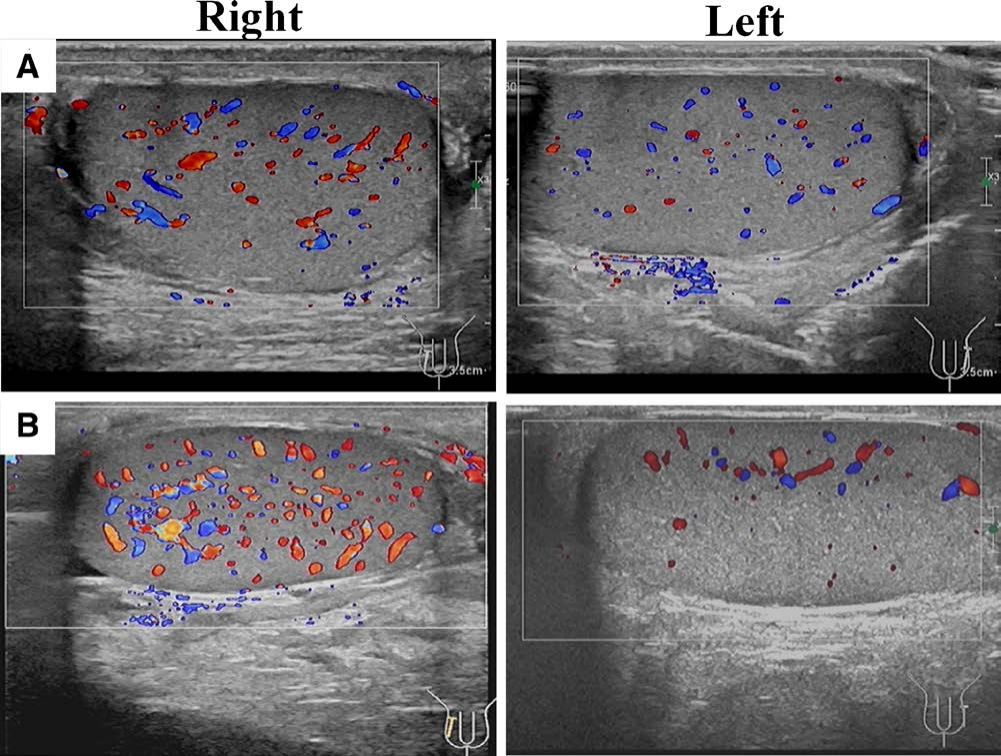
Transscrotal ultrasound imaging in a 34-yr-old patient with acute scrotal pain. (A) Initial color Doppler examination demonstrates markedly increased blood flow in the right testis, with comparative normal blood flow signals observed in the left testis. (B) The next day, an ultrasound reexamination showed that the vascular congestion in the right testicle was significantly increased compared to the left testicle and the previous examination.

Scrotal ultrasound (August 13, 2023): Doppler imaging showed increased blood flow in the right epididymis and testis compared to the left (Fig. 3A). No abscess or torsion was noted. WBC: 9.45 × 10⁹/L (neutrophils 82%) and CRP: 19.65 mg/L. Pyuria (246.5 WBC/µL), hematuria (44 cells per high-power field), and leukocyte esterase.

The patient was started on oral levofloxacin (500 mg daily). However, symptoms persisted 24 hours later, with no reduction in pain or swelling. Repeat ultrasound (August 14, 2023) demonstrated worsening hyperemia in the right testis compared to the prior scan and the left side (Fig. 3B). The regimen was adjusted based on ultrasound results. Levofloxacin switched to intravenous administration (500 mg daily). And cefotaxime (1 g every 12 hours) was added intravenously to broaden antimicrobial coverage. After 10 days of intravenous therapy, the patient’s symptoms resolved completely. Follow-up ultrasound on August 23, 2023, confirmed normalized testis and epididymis.

## 3. Outcomes

Patient 1 underwent anti-inflammatory therapy but developed testicular necrosis, ultimately requiring an orchiectomy. In contrast, patient 2, received antibiotic therapy and achieved a full recovery.

## 4. Discussion

These cases underscore the importance of continuous Doppler ultrasonography to evaluate the symmetry of blood flow, and the changes pre and posttreatment. By integrating real-time hemodynamic feedback into clinical decision-making, clinicians can adjust antibiotic regimens and intensify therapies promptly, thereby mitigating the risk of testicular infarction.

Acute EO represents a common scrotal emergency among adolescents, with early symptoms that are challenging to differentiate from testicular torsion. This condition rarely leads to testicular necrosis.^[4]^ Therefore, prompt diagnosis and appropriate timing of surgical intervention are imperative. Physiologically, the spermatic cord sheath is soft and elastic, assuring unimpeded blood and lymphatic circulation to the testis.^[5]^ Infection-induced congestion and edema lead to thickening of the sheath, compression of venous flow, and testicular engorgement. This compromise can extend to the arterial supply, inciting testicular ischemia, oxygen deprivation, and accumulation of metabolites, potentially culminating in testicular-epidymal infarction if prolonged.

Scrotal color Doppler flow imaging (CDFI) is indispensable for directly assessing testicular blood supply within the established guidelines for EO management.^[6]^ Nonetheless, this guideline does not specify its role in decision-making. Our findings indicate that for effective monitoring, it is essential to maintain consistent follow-up scans with uniform CDFI settings, particularly at brief intervals. Similarly, Wang X et al^[7]^ reported decreased blood flow at the center of the testis and increased flow at the periphery, which may suggest testicular infarction. The first case in this study illustrates the consequences of insufficient vigilance. The 8-day interval between the 2 ultrasound assessments led to a delayed diagnosis of testicular necrosis. Conversely, daily ultrasound surveillance in the second case facilitated timely antibiotic adjustment, forestalling further testicular harm. This difference highlights the role of CDFI as an essential tool in the management of acute orchitis, providing strong support for treatment decisions by closely monitoring changes in testicular blood supply. It has been reported that when a reduction of color Doppler signaling with high intratesticular resistive indices and negative diastolic flow, timely adjustment of the treatment strategy (such as switching to more effective antibiotics or considering surgical exploration) can significantly improve patient outcomes.^[8]^ Our methodology advocates using the contralateral healthy testicles blood flow as an internal control to quantify perfusion abnormalities in the affected side. This approach, combined with pre and posttreatment comparisons, provides an objective framework for evaluating therapeutic efficacy – a strategy not yet standardized in current guidelines.

The contrasting outcomes of the 2 cases presented underscore the critical role of serial Doppler ultrasonography in guiding treatment decisions for acute EO. In Case 1, the 70-year-old patient’s progression to testicular necrosis was likely accelerated by delayed serial Doppler evaluations. Initial imaging on Day 1 revealed increased testicular perfusion, but follow-up scans were not performed until Day 8 (June 11), by which time ischemic changes had already become irreversible. Early and continuous monitoring is crucial in the management of acute EO. The blood flow patterns of the healthy testicle can be used as a baseline to assess the abnormality in the affected side. Dynamic monitoring of blood flow changes, through pre and posttreatment comparisons, can effectively evaluate treatment efficacy. Another critical lesson from Case 1 is the limited predictive value of the improvement in inflammatory markers. Despite the patient’s leukocytosis (WBC: 13.38 × 10⁹/L) and IL-6 levels (678.52 pg/mL) decreasing significantly on June 9, 2024, from initial levels (WBC: 34.41 × 10⁹/L; IL-6: 6129.94 pg/mL) on June 3, the disease worsened, emphasizing that relying solely on blood tests isn’t enough. The third lesson is that patient-specific risk stratification is crucial, especially for individuals with malignancies or cardiovascular disease. It helps to justify the implementation of more aggressive imaging and therapeutic protocols.

In summary, acute EO poses a risk of testicular necrosis, wherein ultrasound serves as an instrumental tool for management. The serial ultrasound examination are pivotal for observing testicular blood flow alterations. It furnishes healthcare professionals with critical data to facilitate timely modifications to the treatment regimen, thereby averting testicular ischemia and necrosis. However, it is critical to acknowledge that the outcomes of CDFI may be subject to operator skill and the sensitivity of the equipment used.^[9]^ To mitigate operator dependency and maintain result consistency, consecutive follow-up examinations should be conducted with the identical ultrasound parameters.

## 5. Conclusion

This study underscores the critical role of serial vascular imaging in guiding treatment decisions for acute EO, particularly in high-risk patients. Meanwhile, these findings emphasize that inflammatory markers such as WBC and IL-6, alone are insufficient to assess treatment efficacy.

## Acknowledgments

Authors are thankful to the urology department for providing the needful information.

## Author contributions

**Data curation:** Chaohong Zhang.

**Formal analysis:** Yuye Wu.

**Investigation:** Bin Cao, Fan Xiao.

**Methodology:** Chaohong Zhang, Fan Xiao.

**Resources:** Yuye Wu.

**Supervision:** Qiufang Ouyang.

**Writing – original draft:** Bin Cao.

**Writing – review & editing:** Qiufang Ouyang.
